# Cases Illustrative of the Benefits from the Use of the Vaginal Speculum

**Published:** 1852-10

**Authors:** E. G. Mygatt

**Affiliations:** Resident of the Society


ART. III.—*Cases Illustrative of the Benefits from the Use of the Vaginal Speculum*, from an address read before the McHenry Co. Medical Society, Aug. 24, 1852, by E. G. MYGATT, M. D., resident of the Society.

CASE I.—Mrs. ——, aged about 32, of a slender constitution, requested my attendance in October, 1849. I found her pale, emaciated, dejected, unable to walk much, or to sit down unless supported by a pillow; great tenderness of the sacrum, and much hypogastric pain and uneasiness; menses regular as to time, but scanty and pale in color. Had complained since her last confinement, some two years previous—not much leucorrhœal discharge. By means of the speculum it was found that the cervix was in its natural position, not much enlarged, but dotted over with superficial ulcerations.

The solid nitrate of silver was applied once a week for 8 or 10 weeks, and injections of warm water were used during the interval. Rest in a recumbent position was also enjoined. This case improved greatly under this treatment, so that the patient was able to attend to her household duties, walk about and visit her neighbors on foot.

Case II.—Mrs. A., near neighbor of the above, asked my advice on the 18th September, 1849. She appeared naturally to have a good constitution, but had been complaining for nearly two years since her last confinement, of hypogastric pains, pruritus and leucorrhœal discharge.

On using the speculum, on the 22nd December, 1849, a fungus was discovered an inch and a half long, the size of a pipe stem, attached to a large ulcerated surface on the anterior lip of the enlarged cervix. The os uteri was extensively ulcerated and turned back towards the rectum. The upper portion of the vagina was red and appeared inflamed and irritated by the muco-purulent secretions with which it was filled. The cervix was thoroughly cauterized with the solid nitrate of silver, once in a week or ten days, varying to avoid the menstrual periods.

In this case cauterization was resorted to twelve times, and during the first few weeks absolute rest and a mild diet were enjoined. The morbid secretions were removed frequently by injections of warm water, and the use of a weak solution of the sulphate of zinc during the intervals. The pruritus was subdued by the following: cor. sub. mur. am. a. a. ʒj., water one pint; apply as a lotion every night and morning. This lotion was very prompt in relieving, not only the mother, but three or four of her daughters, who were, and had been for years affected in the same way.

The patient steadily improved under treatment, and is now in the enjoyment of excellent health.

Case III.—During the month of October, 1849, Mr. ——— called at my office to ask my advice for his wife, whom he represented as affected with severe and long continued dysmenorrhœa and leucorrhœal discharge, of a green appearance. He stated that sexual intercourse was painful, and he feared that she would become deranged if she could not be relieved.

I ascertained by visiting this patient that she was in a miserable state of health, affected with hypogastric pain, cephalalgia, cardialgia, variable appetite, exacerbations of fever, and much mental depression. After the exhibition of suitable medicines to correct the digestive functions, the use of the speculum was resorted to. The cervix uteri was found in its natural position, not much enlarged. The os was open, red, and ulcerated. In the course of the next three months cauterization was resorted to seven times, avoiding the menstrual periods.

The result of treatment in this case has been a decided improvement in the general health, and in the appearance of the os and cervix. The dysmenorrhœa continues in a mitigated form—the paroxysms being shorter and milder.

